# Database of age trajectories of mortality in 110 countries and web application: Data report

**DOI:** 10.3389/fpubh.2022.911589

**Published:** 2022-07-29

**Authors:** Josef Dolejs

**Affiliations:** Department of Informatics and Quantitative Methods, University of Hradec Králové, Hradec Králové, Czechia

**Keywords:** mortality rate, age, data, application software, childhood

## Introduction

The mortality rate is an important indicator of population health. It shows a decline in age after birth and increases with age in older age groups. The increase in adults is approximately exponential and may be interpreted as a manifestation of aging that affects all individuals (1–14).

From an analytical perspective, age remains a deterministic variable in the relationship between age and mortality, and coefficients of determination are higher than 0.99 (4, 10–13). Changes in the mortality rate with age during childhood are quicker, and the steep decline in mortality is accompanied by age-based changes in predominant causes of death (15–20). Congenital anomalies or impairments originating in the perinatal period result in 85% of all deaths during the first 4 weeks of life. However, after the age of 5 years, their contribution reduces to less than 10% (17–20). As such, age is very reliable data as compared to the cause of death.

The author presents the database and web application following the previous studies that included 14 European countries and 25 countries from the four continents (19, 20).

Age trajectories of mortality (ATMs) due to some diseases after birth showed an important phenomenon that may be important in pediatrics. It may be verified or rejected in other regions and countries using the presented database. Age trajectories of total mortality (ATTMs) were the most important ATMs. The decrease in ATTMs was described by the model of the inverse proportion with coefficients of determination higher than 0.99 (15, 17, 19, 20).

The ATMs as a result of major disease groups were decreased with age after the first year of life, with one exception, malignant neoplasms (15, 17, 19, 20). The ATM due to neoplasms was declined only during the first month of life and remained age-independent from the first month till the age of 20 years.

Further, ATMs due to major disease groups (e.g., “neoplasms” or “infectious diseases”) were composed of a larger spectrum of diseases. The database enables researchers to investigate the composition of ATM (the spectrum of diseases that contributed to some ATM). Researchers or other users may combine diseases in a selected population, and the resulting ATM will be transferred to the web application. The results shown in the previous studies may be verified or rejected using the database in 110 countries and in 10 aggregated populations.

## Methods

### World health organization raw data

The WHO Raw Mortality database was the main source of data (21). It contains the number of deaths within specific age categories in different countries (21).

The cause of death was determined using the specific revision of the International Classification of Diseases (ICDs) in the database. The database contains the 7th−10th revisions of ICD. Specific revisions were used by different countries at different time periods. The use of the 10th revision of ICD (ICD10) was the first main criterion to segregate data (22). The number of deaths due to causes classified as per ICD10 was found in two files in 2019 (21).

The database does use different categories for the age that differ mainly during the first year of life. The following four age categories in the first year of life were the most detailed: 0–24 h after birth, 1–7, 7–28, and 28–365 days. Not all countries use these four categories, and hence, the second criterion for segregating data was the four age categories.

**Figure 1:**
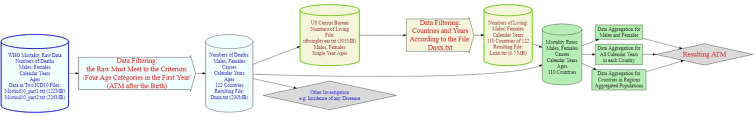
Diagram of the process.

Thus, data using ICD10 were selected, and the resulting two files “Morticd10_part1.txt” and “Morticd10_part2.txt” were aggregated to create one file. Within this, the rows with four age categories in the first year were used as filters for segregation. Figure 1 is a diagrammatic representation of this process.

Applying the ICD10 and age criteria reduced the number of countries to 122. The number of calendar years in some countries were reduced too. The WHO database uses the four mentioned age categories in the first year of life, a single age category for the age range 1–5 years and ages above 5 years are grouped with an interval of 5 years, each up to ages 90–94 years.

The next important step was sourcing data on the number of people alive. WHO database contains the number of living people in the file “pop.txt” but it uses one age category in the age range 1–5 years in some countries in some years. For this reason, the set of years does not reach the set of years used in the two files “Morticd10_part1.txt” and “Morticd10_part2.txt”. For example, only the years 1966–1989 may be found in the US with single-year age categories. The “United States Census Bureau” database of living people in a single-year age group may solve the problem, and it was used to automate the process of ATM construction (23). A total of 110 out of 122 countries were available in the database, and thus, the ATM was constructed for these 110 countries.

Further, 10 large, aggregated populations that contained countries from different regions of the world were created. The largest aggregated population “P110” contained all the 110 countries. The number of people alive per 1 year in “P110” was approximately 2.286 billion, and it represented about one-third of the whole world population during the study period. The list of 110 countries may be shown in the web application if an aggregated population is selected in the panels: “ATM2,” “CACNS,” and “Main Chapters.”

### Number of people alive—united states census bureau

The database “United States Census Bureau” enabled us to download file “idbsingleyear.txt” that included the number of people alive (men and women) in 110 countries, in 1-year age categories, and in calendar years (23). The set of 122 countries and the set of calendar years in each country were used to find the suitable number of people alive (men and women) in the file “idbsingleyear.txt.” The process reduced the number of countries from 122 to 110. Three regions, “*United Kingdom England and Wale*s,” “*United Kingdom, Northern Ireland*,” and “*United Kingdom, Scotland*,” were not used separately as all three regions were referred to as “*United Kingdom*” in the file “idbsingleyear.txt.” Further, nine countries were not found in the file “idbsingleyear.txt,” and they were excluded from the list of 122 countries: “*Mayotte,” “Reunion,” “Rodrigues,” “French Guiana,” “Guadeloupe,” “Martinique,” “Netherlands Antilles,” “Occupied Palestinian Territory,” and “Serbia and Montenegro”* (Figure 1). The number of people alive in the first age category was used to calculate the ATM in the four age categories as categorized by WHO for the first year of life. The next four single-year age categories were used to calculate ATM in the age range 1–5 years. Similarly, the 5-year age categories (from ages 5 to 95) were calculated from the single-year age categories.

### Halley method—aggregation of calendar years and countries

The 1-year period is a typical time unit used in epidemiology, biology, and social sciences. Biological events (such as death or disease) in different ages may be studied in one specific calendar year across age categories (cross-sectional description). The other possibility is the longitudinal observation of individuals born in the same year (generation study or longitudinal study). If the time unit “1 year” was replaced with the time unit “1 day” (e.g., for some biological, social, or cultural reasons), events observed for 1 year represented the aggregation of the events that occurred in 365 days. The aggregation of more calendar years had a similar meaning. Age was assumed as the main factor, and all other factors were assumed to be less significant. Furthermore, the existence of general mechanisms was assumed, as demonstrated in the ATM after birth. For these reasons, ATM may be constructed in as large a population as possible (19, 20). Including more regions and more calendar years within the analysis may eliminate all factors other than age, rendering the impact of age more visible. The following standard definition of the force of mortality at age x was used:


(1) $$\mu (x)={\text{lim}}_{h\to 0}\frac{D(x+h)}{L(x)}=-\frac{\frac{dS(x)}{dx}}{S(x)}≅\frac{Di}{Li}.\frac{1}{\left(Bi-Ai\right)}$$

where *D*(*x*+*h*) is the number of deaths in a small age range, [x, x+h), the infinitesimal increment *h* is positive. L(x) is the number of living people at the age x, *S*(*x*) is the survival function (percentage of living people at age *x*), which is valid in principle: *S*(*x*) = 1 – *F*(*x*), where *F*(*x*) is the cumulative distribution function of the probability of death. The empirical value *Di* is the number of deaths within an age range [*Ai, Bi*), while *Li* is the size of the population among which the deaths occurred. Empirically, changes in *Li* within an age interval [*Ai, Bi*) were very small in childhood, when compared with changes in *Di*, and the number of living people in *Li* within an age interval [*Ai, Bi*) was used instead of the average number of living people in the region and calendar period. Namely, population *Li* went through the “window” in time, and it was the meaning of the product *Li*•(*Bi* – *Ai*) in Equation (1). Element Bi – Ai differed from 1 only in the first four age categories where the four values, 1/365 years; 6/365 years; 21/365 years; and (365–28)/365 years, were used. It equaled to 1 in the 5-year age categories because Di and Li were detected in one calendar year. The unit corresponding to the mortality rate was “person-years,” which meant the number of years lived by members of the population between the ages *Ai* and *Bi*. Uncertainty or possible demographic error of Li was discussed in the previous studies in detail and they were negligible with the respect to resulting ATM (11–13, 17–20).

The ATMs were assumed to be unknown theoretical curve and was constructed using the right side of the equation (1). Mortality rates in different age groups described the group's Li in the same way a decay constant may describe the force of a radioactive decay on different radionuclides (the different radionuclides correspond to the group's Li in the parallel) (19, 20, 24).

Zero deaths in a given age group may be the major problem for the construction of ATM from a specific disease or from a set of diseases. While zero deaths due to some disease may occur within a specific age category, in a specific calendar year, at least one death may occur within the same age category in other calendar years. The inclusion of additional calendar years may remove this obstacle. The method was first utilized by the well-known astronomer and mathematician Edmond Halley (25). This method enabled the calculation of the mortality rate within one age category based on the number of deaths and living persons in several years (25–27). Standard epidemiologic and demographic interpretations of the specific value of mortality rate within a specific age category differed from the values calculated for one calendar year. However, the interpretation was the same for all age categories, and the resulting ATM may be interpreted as a hallmark of aging. In addition, including more regions and calendar years may eliminate factors other than age.

The ATM in one country in one calendar year may be constructed separately for men and women using three main data files, which are described in the paragraph “Three Main Data Files.”

### Aggregated populations

In total, 10 aggregated populations were mainly created to eliminate zero cases due to specific diseases. The aggregated population may be selected by the user in the web application in the panels “ATM2,” “CACNS,” and “Main Chapters.” A list of countries in the aggregated population is automatically shown in the panels.

The largest aggregated population “***P110***” contained all 110 countries. “***P28EU***” contained 28 European countries, “***P42SCA***” contained 42 countries from South and Central America, and “***P6AS***” contained six Asian countries. Three groups of European countries from the previous study were used in the aggregated populations: “***P5BigEU*,**” “***P5SmallEU*,**” and “***P4NordEU*.**” (19) Aggregated population “***P6SA***” contained six countries of South America (20). In addition, two aggregated populations “***P14***” and “***P25***” used similar to their use in the previous studies (“***P14***” = “***P5BigEU***” + “***P5SmallEU***” + “***P4NordEU***” and “***P25***” = “***P14***” + “***P6SA***”) (19, 20).

Calendar years used by a specific country in the database present in the web application if a specific country is selected in the panels “ATM2,” “CACNS,” and “Main Chapters” (the list found in the file as “CountriesDnxx.txt”).

### Three main data files

**Figure 2:**
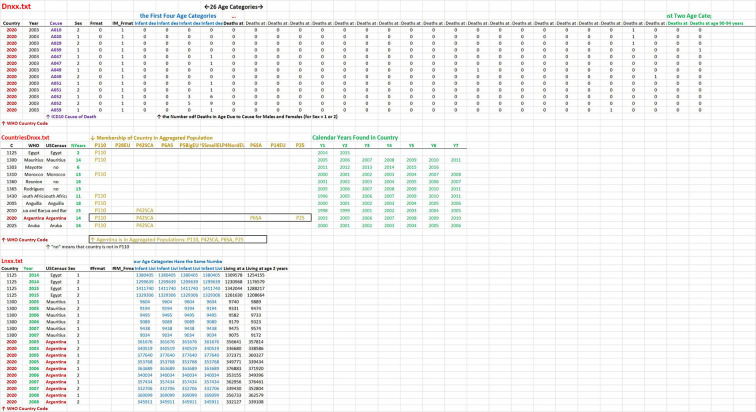
Structure of three main files. The structure of the two files “Dnxx.txt” and “Lnxx.txt” is similar. Each row contains the numbers of deaths in “Dnxx.txt” and the numbers living in “Lnxx.txt,” in a specific calendar year, for men and women separately in age categories. The first two columns contain country codes and calendar years in both files. The third column contains the cause of death in “Dnxx.txt” while the country name in “Lnxx.txt.” The other columns have the same meaning in the two files. The fourth column contains sex, the next two columns contain an index of WHO format of age categories, and the last columns represent 26 age categories (columns: 7, 8, …32). The file “ContriesDnxx.txt” contains information about a specific country. The columns contain the WHO code, the name of the country used in WHO, the name of the country used in the US Census Bureau, and the number of calendar years. Columns 5–14 contain the indication if the country was used in aggregated population (a specific column corresponds to a specific aggregated population). The last columns contain the list of calendar years found in the specific country in the WHO database.

The three main data files, “Dnxx.txt,” “CountriesDnxx.txt,” and “Lnxx.txt,” may be downloaded in the web application in the panel “Introduction.” The structure of the files is shown in Figure 2.

The data may be obtained in different ways than in the web application. For example, in the aggregation of calendar years, men and women may be ignored. In this case, ATM in the specific calendar year may be constructed using the files “Dnxx.txt” and “Lnxx.txt” (e.g., in Excel). Calendar years and age categories were unified in “Dnxx.txt” and “Lnxx.txt.” Users may calculate ATM using the formula (1) (the element Bi-Ai differs from the value of one in the first four age categories).

## Data investigation and web application

There are more possibilities on how to run the web application:

(a) Directly on the Internet: https://atm-dolejs-josef.shinyapps.io/ATTM/
(b) Download the directory from the Supplementary Material
(c) Download the directory here https://lide.uhk.cz/fim/ucitel/dolejjo1/

If the directory is downloaded, the application may run according to the instructions in the file “Run Application in Personal Device with Windows.pdf.”

The application contains 10 panels: Introduction, ATTM1, ATTM2, CACNS, Main Chapters, Detail Codes1, Detail Codes2, Combinations of Main Chapters, Significance of Main Chapters in Age, and Other Models.

The first panel “Introduction” contains an introduction to ATM after the birth and a short explanation of the theory of congenital individual risks of death (TCIR) (16, 17, 19, 20). The panel contains three keys to download three main files “Dnxx.txt,” “DnxxCountries.txt,” and “Lnxx.txt,” the fourth button to download the file “TCIR.pdf” with the explanation of TCIR, and fifth key to download the file “Numerical_Bending_ATM_in_TCIR.xlsx” with the demonstration of bending ATM according to TCIR (16, 17, 19, 20).

The second panel “ATTM1” contains an illustrative animation of ATTM in 110 countries and in 10 aggregated populations. The panel “ATTM2” contains the same plot, while each population may be individually selected. Moreover, a list of all countries and the mean number of people alive in all age categories in specific calendar years in the selected aggregated population is shown. The two identical plots in ATTM1 and ATTM2 contain coefficient of determination calculated in the model of the inverse proportion in the age range 0–10 years (in the first nine points). Confidence bands are common for all populations. They were calculated as limits between which 95% of empirical values, in each age category, were detected in the 110 countries.

The panel “CACNS” is similar to the panel “ATTM2”; however, ATM due to congenital anomalies of the central nervous system (CACNS) is shown. The age range used in the model of the inverse proportion with the 95% confidence bands is 0–40 years.

In the panel “Main Chapters,” one aggregated population and one main chapter of ICD10 (one of 22) may be selected. The ATM plot using the selection of the main chapter in the chosen aggregated population with four illustrative straight lines is displayed. Two straight lines have the slope −1 and go exactly through to the second point (the first week) and through to the fifth point (1–2 years), respectively. One straight line has the slope −2 and goes through the fifth point. The last straight line has a slope 0 and corresponds to the age independence. These straight lines may be important and may be used to roughly classify ATM (the straight lines are not the regression lines but they have an exact slope and go through observed mortality rates in specific age categories). In addition, the spectrum of ATMs that are significant to ATTMs are shown in the panel below in two plots, in two age ranges “0–10 years” and “1–10 years.”

The aggregated population and main chapter of ICD10 selected in the panel “Main Chapters” are used automatically in the panel “Detail Codes1.” The resulting list of all disease codes is displayed. The users may select their own set of codes in the section “Choose Diseases of ICD10” and create a specific group of diseases. The ATM due to the group of diseases is displayed with four indicative straight lines (the same four straight lines as described in the panel “Main Chapters”). There are two keys to download data files with the list of selected codes and with the ATM data as per the disease groups.

Three illustrative examples of the use of the panel “Detail Codes1”:

(a) It was shown that ATMs due to the second chapter of ICD10 (Neoplasms) were age independent in the age range from the first month to 25 years (16, 17, 19, 20). If the aggregated population is chosen in the panel “Main Chapters,” the list of all ICD10 codes used in all age categories will be seen. Each code may be selected by a click on the left panel (it may be removed by a click on the right panel). The ATM due to the selected set of codes is calculated, and a plot is shown (two possible levels of ICD10 were used in the WHO database, and the problem is described below).
(b) It was shown that ATMs due to the first chapter of ICD10 (and due to other chapters) were age independent or declined slowly with age in the first year of life. Simultaneously, the decrease with age was faster after the first year (usually with the slope −1) (16, 17, 19, 20). The bending ATMs were typical for many main chapters of ICD10 in which congenital impairment was not determined as the cause of death. It is possible to study the spectrum of diseases in the selected main chapter and in the selected aggregated population.
(c) It was shown that ATMs due to CACNS (Q00–Q07, congenital malformations of the central nervous system) were inversely proportional to age in higher age categories (e.g., 40 years) (16–20). If the aggregated population and the 17th chapter of ICD10 (all congenital anomalies) are selected in the panel “Main Chapters,” then the study similar to the previous two examples “a, b” may be accomplished (only codes Q00–Q07 may be selected).

Some basic models may be tested directly in the panel “Detail Codes1,” and ATM may be downloaded as a data file for other investigations.

The panel “Combinations of Main Chapters” enables to create any combination of main chapters of ICD10 in the selected aggregated population. For example, the contribution of the main chapters to ATTM may be studied here. Key “Downloaded Mortality Rates to CSV File” in the panel “Combination of Main Chapters” may be used to download mortality rates for the selected main chapter in the selected aggregated population.

Proportions of main chapters in selected aggregated population in age categories are shown in the panel “Significance of Main Chapters in Age.” Primarily, it shows that the significance of cases with congenital impairments (the 16th and the 17th chapter of ICD10) dramatically decreases with age. The special group of diseases “Other Diseases” contains the first 15 chapters of ICD10, excluding the second chapter of ICD10 (Neoplasms) (16, 17, 19, 20).

Historical models found in the literature, which may describe ATTM after the birth, are displayed in the last panel “Other Models.” (11–13).

## Data availability statement

The original contributions presented in the study are included in the article/supplementary material, further inquiries can be directed to the corresponding author/s.

## Author contributions

The author confirms being the sole contributor of this work and has approved it for publication.

## Funding

The paper was written with the support of the specific project 6/2022 grant-Determinants of Cognitive Processes Impacting the Work Performance granted by the University of Hradec Králové, Czech Republic and thanks to help of student Martina Janeckova. It was written also with the support of the Specific Research Project of the Faculty of Informatics and Management of University of Hradec Králové in 2022 was kindly acknowledged.

## Conflict of interest

The author declares that the research was conducted in the absence of any commercial or financial relationships that could be construed as a potential conflict of interest.

## Publisher's note

All claims expressed in this article are solely those of the authors and do not necessarily represent those of their affiliated organizations, or those of the publisher, the editors and the reviewers. Any product that may be evaluated in this article, or claim that may be made by its manufacturer, is not guaranteed or endorsed by the publisher.
