# Supplementary material for: Database of age trajectories of mortality in 110 countries and web application: Data report
Source: Front Public Health. 2022 Jul 29;10:911589. doi: 10.3389/fpubh.2022.911589 (PMC9374568; doi:10.3389/fpubh.2022.911589)
Supplement: Supplementary file 1 [file Data_Sheet_1.zip › ATM_Dolejs/Run Application in Personal Device with Windows.docx]

**Run Application in Personal Device with Windows 10+ without Internet Connection**

1. Download and copy the whole directory "***ATM_Dolejs***" to your device (e.g. "***C:\ ATM_Dolejs***").
   e.g. here <https://lide.uhk.cz/fim/ucitel/dolejjo1/> click the button and open downloaded zip file.
2. Install previous version of "***R***" in your device:
   download the installation file "[**R-4.1.0-win.exe**](https://cran.r-project.org/bin/windows/base/old/4.1.0/R-4.1.0-win.exe)" here :
   **https://cran.r-project.org/bin/windows/base/old/4.1.0/**double-click the downloaded file to run the installer and confirm standard settings.
3. Install previous version of "***RStudio***" in your device:
   download the installation file "[**RStudio-pro-1.4.1717-3.exe**](https://download1.rstudio.org/desktop/windows/RStudio-pro-1.4.1717-3.exe)" here :
   **https://dailies.rstudio.com/version/1.4.1717-3/**double-click the downloaded file to run the installer and confirm standard settings**.**
4. Run ***RStudio:***use standard shortcut or click on the file "***rstudio.exe***" in the subdirectory
   ***"C:\Program Files\RStudio\bin"***.
5. Install the following five packages in ***RStudio***:
   select the ***Packages tab*** in the lower-right pane and press the ***Install button,***copy here names of the packages with comma to the opened panel
   ***shiny, shinythemes, shinyWidgets, propagate, ggplot2***and press "***Install***" (wait for some minutes).
6. In ***RStudio***, open the web application with the file "***app.R***":
   select "***File***" in menu, select "***Open File***" and find out the file "***app.R***" in the dialog window
   in the directory "***ATM_Dolejs***", and press "***OK"***.
7. Run web application in ***RStudio***:
   click the button "***Run App***" on the right corner in the main window in ***RStudio***,
   (if web application is opened in ***RStudio***), press ***Open in Browser*** in web application,
   change **zoom in browser as necessary**.
