# Supplementary material for: Database of age trajectories of mortality in 110 countries and web application: Data report
Source: Front Public Health. 2022 Jul 29;10:911589. doi: 10.3389/fpubh.2022.911589 (PMC9374568; doi:10.3389/fpubh.2022.911589)
Supplement: Supplementary file 1 [file Data_Sheet_1.zip › ATM_Dolejs/www/Equations.docx]

**Equations in Theory of Congenital Individual Risks (TCIR)**

The decrease of mortality rates with age after the birth is caused by the depletion of individuals with more sever congenital impairment. Possible changes of the impairments with age are negligible if compared with the variability of congenital impairments (congenital anomalies and impairments originated in the perinatal period). Each congenital individual risk is assumed to be age independent if compared with the whole spectrum of congenital impairments in the whole born population. A subpopulation is characterized by the congenital individual risk of death r and by the initial number of born children L_o_(r). The subpopulation is reducing during age x and number of living at age x is:

${L\left( r,x \right)=L}_{o}\left( r \right).exp(-r.x)$ (1)

If continuous formalism is used, the number of all living children at age x is the integral (or the sum) of all r values:

$L\left( x \right)=\int_{0}^{\propto} L\left( r,x \right).dr=\int_{0}^{\propto} L_{o}\left( r \right).\exp\left( -r.x \right).dr$ (2)

Mortality rate in the whole population in age x using the assumption (1) is:

$\mu\left( x \right)=-\frac{\frac{\partial L\left( x \right)}{\partial x}}{L\left( x \right)}=-\frac{\int_{0}^{\propto} -r.L_{o}\left( r \right).\exp\left( -r.x \right).dr}{\int_{0}^{\propto} L_{o}\left( r \right).\exp\left( -r.x \right).dr}$ (3)

Since the empirical changes of L(x) are very small, if compared to the empirical changes of the numbers of deaths (D(x)), the denominator may be replaced by the number of all born children Lo. Empirically, L(x) varies less than 2%, while the numbers of deaths D(x) varies in the magnitude of more than three orders during the first 20 years. Consequently, the following is valid:

$\mu\left( x \right)\cong\frac{\int_{0}^{\propto} r.L_{o}\left( r \right).\exp\left( -r.x \right).dr}{L_{o}}=\int_{0}^{\propto} r.f\left( r \right).\exp\left( -r.x \right).dr$ (4),

where f(r) is the density function of r at the moment of birth. It may be interpreted by the following formula:

$f\left( r \right)=\frac{L_{o}\left( r \right)}{L_{o}}=\frac{L_{o}\left( r \right)}{\int_{0}^{\propto} L_{o}\left( r \right).dr}$ (5)

The denominator in the equation (5) corresponds to the number of all born people L_o_.

Mathematically, mortality rate in the whole population at age x is the Laplace transform of the product r.f(r) in the equation (4). For example, if f(r) is the density function of the log-normal distribution or if the rule “the more severe the impairment, the less frequently it occurs in the born population” (the relationship f (r) ≅ constant/r) is valid, mortality rate is given by:

$\mu\left( x \right)\cong\int_{0}^{\propto} c.\exp\left( -r.x \right).dr=c.\left[ \frac{\exp\left( -r.x \right)}{-x} \right]_{0}^{\infty}=\frac{c}{x}$ (6)

It was also shown that, if f(r) is the density function of normal distribution with big variation, or if f(r) is approximately constant in an important interval, mortality rate is given by:

$\mu\left( x \right)\cong\int_{0}^{\propto} r.c.\exp\left( -r.x \right).dr=c.\left[ \frac{-r.\exp\left( -r.x \right)}{-x}-\frac{exp(-r.x}{\left( -x \right)^{2}} \right]_{0}^{\infty}=\frac{c}{x^{2}}$ (7)

What does "an important interval of r" mean? The theoretical range of r is from zero to infinite, but it is clear that, empirically, it should range between limited values (r_min_, r_max_). For example, if r_max_ is about 1000 and r_min_ is about 0.0001 then the formulas (6) and (7) are numerically valid within the age range [0, 20) years (it follows from simple numerical calculations). In other words, the equations (5) and (6) are weakly affected by the majority of the population with very low r, and shape of ATM is determined by small subpopulations with higher values of r. Consequently, the assumptions about distribution of the r value are, in fact, related only to a small part of the born population with higher r values. For example, the assumption "the more severe the impairment, the less frequently it occurs in the born population" is, in fact, related to the subpopulation with higher r values (this is not true for ATM due to CACNS because the age range may be wider and relatively smaller values of r may be important). In a simplified way, lower ages correspond to higher values of r while higher ages correspond to lower values of r in the formalisms.

**Other historical models of ATM after the birth**

The first historical model of ATM after the birth was formulated by **Thiel in 1871**.**^1^** He proposed simple exponential decrease of mortality with age. It is concave in the log-log scale and the model was the first term in the following general formula describing all ages:

$\mu\left( x \right)=A.e^{-B.x}\text{ + }{C.e}^{{D.\left( x-E \right)}^{2}}+F.e^{G.x}$ (8)

The formula had tried to describe age trajectory of total mortality from birth up to high ages and the first element is labeled here as "**Exp**".

Other group of studies had tried to describe the mortality changes with age in the narrower age interval [1, 12) months.**^2-5^** The studies used the following formula for cumulative deaths q(n) up n months:

$q\left( n \right)=a+b.\left[ \ln\left( n+1 \right) \right]^{3}$ for 1 month ≤n ≤ 12 months (9)

The model (9) may be formulated for mortality rate μ(x) at age x:

$S\left( x \right)=1-\frac{q\left( x \right)}{Lo}=1-\frac{a+b.\left[ \ln\left( 12.x+1 \right) \right]^{3}}{Lo}$ (10)

$\mu\left( x \right)=-\frac{\frac{dS\left( x \right)}{dx}}{S\left( x \right)}=-\frac{\frac{d\left\{ 1-\frac{a+b.\left[ \ln\left( 12.x+1 \right) \right]^{3}}{Lo} \right\}}{dx}}{\left\{ 1-\frac{a+b.\left[ \ln\left( 12.x+1 \right) \right]^{3}}{Lo} \right\}}$ (11)

$\mu\left( x \right)=\frac{\left\{ \left( \frac{b}{Lo} \right).\frac{d\left[ \ln\left( 12.x+1 \right) \right]^{3}}{dx} \right\}}{\left\{ 1-\frac{a+b.\left[ \ln\left( 12.x+1 \right) \right]^{3}}{Lo} \right\}}$ (12)

The model (12) is labeled here as "**BP**". Additionally, **Heligman and Pollard** proposed a general relationship for the age all‑causes mortality in 1980, and the following term was suggested for the decline after birth:**^6,7^**

$\mu\left( x \right)=A^{\left( x+B \right)^{C}}\text{ }\text{, for}\text{: 0 < A < 1, 0 < B < 1, 0 < C < 1}$ (13)

The model (13) is also concave in the log-log scale and it is labeled here as "**HP**".

Besides the three models, the **Weibull model (WM)** with two parameters is linear in the log-log scale, and may be taken into account. **WM** has strong assumption that the absolute value of slope should be less than 1. It is valid for the distribution function F(x) of ages of died people in **WM**:

$F\left( x \right)=1-S\left( x \right)=1-e^{\left( \frac{-x^{m}}{a} \right)}$ for a > 0 and m > 0 (14)

Furthermore, mortality rate μ(x) at age x is:

$\mu\left( x \right)=-\frac{\frac{dS\left( x \right)}{dx}}{S\left( x \right)}=-m.\left( \frac{-x^{m}}{a} \right).\frac{e^{\left( \frac{-x^{m}}{a} \right)}}{e^{\left( \frac{-x^{m}}{a} \right)}}=-m.\left( \frac{-x^{m}}{a} \right)=\mu_{1}x^{m-1}$ (15)

Slope equals to **m -1** in the log-log scale and parameter **m** is zero if **the inverse proportion is valid**. If **m** Ł**0** (if slope Ł -1) then F(x) **does not increase with age x and it is not the distribution function**. Consequently, **WM** may not be used if slope m-1Ł -1. If the mortality decrease is not very steep and m-1 > -1 then the linear model in the log-log scale corresponds to the Weibull distribution.

**References**

1. Vaupel, J. W.; J. R. Carey, K. Christensen; T. E. Johnson; A. I. Yashin; N. V. Holm; I. A. Iachine; et al. 1998. Biodemographic trajectories of longevity. Science 1998 280:855–860.

2. Hayflick L. Aging is not a disease. Aging. 1998; 10 (2): 146.

3. Halley E. An estimate of the degrees of mortality of mankind, drawn from curious tables of the births and funerals at the city of Breslaw, with an attempt to ascertain the price of annuities on lives. Philosophical Transactions. 1693; 17:596 610.

4. Bellhouse DR. A new look at Halley’s life table. J. R. Statist. Soc. A. 2011; 174(3): 823 832.

5. Luy MA, Wittwer-Backofen U. The Halley Band for Paleodemographic Mortality Analysis. Recent Advances in Palaeodemography. 2008; 119 141.

6. Heligman L, Pollard JH. The Age Pattern of Mortality. Journal of the Institute of Actuaries. 1980; 107(1):49 75.

7. Preston SH, Heuveline P, Guillot M. Demography: measuring and modeling population processes. Oxford, Blackwell. 2001; 190 194.
