# Supplementary material for: Database of age trajectories of mortality in 110 countries and web application: Data report
Source: Front Public Health. 2022 Jul 29;10:911589. doi: 10.3389/fpubh.2022.911589 (PMC9374568; doi:10.3389/fpubh.2022.911589)
Supplement: Supplementary file 1 [file Data_Sheet_1.zip › ATM_Dolejs/www/Historical models.pdf]

## Other historical models of ATTM after the birth

The first historical model of ATM after the birth was formulated by **Thiel in 1871**.[1] He proposed simple exponential decrease of mortality with age. It is concave in the log-log scale and the model was the first term in the following general formula describing all ages:

$$\mu(x) = A.e^{-B.x} + C.e^{D.(x-E)^2} + F.e^{G.x} \tag{1}$$

The formula had tried to describe age trajectory of total mortality from birth up to high ages and the first element is labeled here as "**Exp**".

Other group of studies had tried to describe the mortality changes with age in the narrower age interval [1, 12) months.[2-5] The studies used the following formula for cumulative deaths q(n) up n months:

$$q(n) = a + b.[\ln(n+1)]^3 \qquad \text{for 1 month} \le n \le 12 \text{ months} \tag{2}$$

The model (9) may be formulated for mortality rate μ(x) at age x:

$$S(x) = 1 - \frac{q(x)}{Lo} = 1 - \frac{a+b.[\ln(12.x+1)]^3}{Lo} \tag{3}$$

$$\mu(x) = -\frac{dS(x)}{dx}\Big/S(x) = -\frac{d\left\{1-\frac{a+b.[\ln(12.x+1)]^3}{Lo}\right\}}{dx}\Bigg/\left\{1-\frac{a+b.[\ln(12.x+1)]^3}{Lo}\right\} \tag{4}$$

$$\mu(x) = \left\{\left(\frac{b}{Lo}\right).\frac{d[\ln(12.x+1)]^3}{dx}\right\}\Bigg/\left\{1-\frac{a+b.[\ln(12.x+1)]^3}{Lo}\right\} \tag{5}$$

The model (5) is labeled here as "**BP**". Additionally, **Heligman and Pollard** proposed a general relationship for the age all-causes mortality in 1980, and the following term was suggested for the decline after birth:[6,7]

$$\mu(x) = A^{(x+B)^C} \quad , \text{for: } 0 < A < 1,\ 0 < B < 1,\ 0 < C < 1 \tag{6}$$

The model (6) is also concave in the log-log scale and it is labeled here as "**HP**".

Besides the three models, the **Weibull model (WM)** with two parameters is linear in the log-log scale, and may be taken into account. **WM** has strong assumption that the absolute value of slope should be less than 1. It is valid for the distribution function F(x) of ages of died people in **WM**:

$$F(x) = 1 - S(x) = 1 - e^{\left(-x^m/a\right)} \qquad \text{for } a > 0 \text{ and } m > 0 \tag{7}$$

Furthermore, mortality rate μ(x) at age x is:

$$\mu(x) = -\frac{dS(x)/dx}{S(x)} = -m.\left(-x^m/a\right).e^{\left(-x^m/a\right)}\Big/e^{\left(-x^m/a\right)} = -m.\left(-x^m/a\right) = \mu_1 x^{m-1} \tag{8}$$

Slope equals to **m -1** in the log-log scale and parameter **m** is zero if **the inverse proportion is valid**. If **m ≤0** (if slope ≤ -1) then F(x) **does not increase with age x and it is not the distribution function**. Consequently, **WM** may not be used if slope m-1≤ -1. If the mortality decrease is not very steep and m-1 > -1 then the linear model in the log-log scale corresponds to the Weibull distribution.


### References

1. Vaupel, J. W.; J. R. Carey, K. Christensen; T. E. Johnson; A. I. Yashin; N. V. Holm; I. A. Iachine; et al. 1998. Biodemographic trajectories of longevity. Science 1998 280:855–860.
2. Hayflick L. Aging is not a disease. Aging. 1998; 10 (2): 146.
3. Halley E. An estimate of the degrees of mortality of mankind, drawn from curious tables of the births and funerals at the city of Breslaw, with an attempt to ascertain the price of annuities on lives. Philosophical Transactions. 1693; 17:596 610.
4. Bellhouse DR. A new look at Halley's life table. J. R. Statist. Soc. A. 2011; 174(3): 823 832.
5. Luy MA, Wittwer-Backofen U. The Halley Band for Paleodemographic Mortality Analysis. Recent Advances in Palaeodemography. 2008; 119 141.
6. Heligman L, Pollard JH. The Age Pattern of Mortality. Journal of the Institute of Actuaries. 1980; 107(1):49 75.
7. Preston SH, Heuveline P, Guillot M. Demography: measuring and modeling population processes. Oxford, Blackwell. 2001; 190 194.
