# Supplementary material for: Database of age trajectories of mortality in 110 countries and web application: Data report
Source: Front Public Health. 2022 Jul 29;10:911589. doi: 10.3389/fpubh.2022.911589 (PMC9374568; doi:10.3389/fpubh.2022.911589)
Supplement: Supplementary file 1 [file Data_Sheet_1.zip › ATM_Dolejs/www/Relationships describing bending ATM.pdf]

Simple explanation of bending ATM according to "*Theory of congenital individual risks*" *(TCIR)*.

As the present project aimed to describe the dynamics of the relationship between age and mortality, we utilized the following definition of the force of mortality at exact age *x*:

$$\mu(x) = \lim_{h\to 0} \frac{D(x+h)}{L(x)} = -\frac{dS(x)}{dx} \Big/ S(x) \cong \frac{Di}{Li} \cdot \frac{1}{(Bi-Ai)} \quad , \qquad (1)$$

where $D(x+h)$ is the theoretical number of deaths in a small age interval [x, x+h), the theoretically infinitesimal increment *h* is positive, and age approaches exact age *x* "from the left". $S(x)$ represents the survival function (percentage of living people at age *x*), which is valid in principle: $S(x) = 1-F(x)$, where $F(x)$ is the cumulative distribution function of the probability of death. The empirical value *Di* represents the number of deaths in a specific age interval [*Ai*, *Bi*), while *Li* represents the size of the population among which the deaths occurred. Changes in *Li* within an age interval [*Ai*, *Bi*) are empirically very small when compared with changes in *Di*. For this reason, the number of living people *Li* at age *Ai* can be used instead of the average number of living people. In other words, population *Li* goes through the "window" in time or through the age interval [*Ai*, *Bi*). The product *Li*•(*Bi*-*Ai*) in equation (1) represents the number of "person-years", or the number of years lived by members of the population between ages *Ai* and *Bi*. The age trajectory of mortality is assumed to be an unknown theoretical curve, and it is constructed using the right side of equation (1). Mortality rate at an exact age, force of mortality, or simply mortality rate describes different age groups similarly to the way a decay constant describes the force of radioactive decay on different radionuclides. (In this comparison, the different radionuclides correspond to the various groups of patients.)

If some maximal limit bounds congenital individual risks in the born population, then the following approximation may be valid according to the TCIR (Dolejs, 2001; Dolejs, 2003):

$$\mu(x) = \int_0^{r_{max}} c.e^{(-r.x)} dr = c.\left[\frac{e^{(-r.x)}}{-x}\right]_0^{r_{max}} = c.\frac{e^{(-r_{max}.x)}}{-x} - c.\frac{1}{-x} = \frac{\mu_1}{x}.\left[1 - e^{(-r_{max}.x)}\right] \qquad (2)$$

Number of deaths in age categories *Di* decreased by three orders of magnitude, and percentage of living $S(x)$ was higher than 0.98 at the age of 10 years. Consequently, the changes in the denominator in formula (1) (number of living persons) with age can be neglected due to the changes in the numerator of equation (1). Based on the TCIR, we simultaneously assumed that the distribution of individual congenital risk *r* can be approximated by the relationship $f(r) = c/r$. Because the numerator is the partial derivative of $S(x)$ with respect to the variable *x*, the variable *r* is only in the exponential term. (Variable *r* represents the denominator in the frequency function $f(r)$, and it is also the result of the partial derivative in the numerator.) Furthermore, if the product $r_{max}.x$ is small in equation (2), then the theoretical mortality rate is approximately constant according to the following relationship (3) (Dolejs, 2001; Dolejs, 2003):

$$\mu(x) = \frac{\mu_1}{x}.\left[1 - e^{(-r_{max}.x)}\right] \cong \frac{\mu_1}{x}\left[1 - (1 - r_{max}.x)\right] = \mu_1.r_{max} \qquad (3)$$

If a time unit of 1 year is used, and if r is represented per year, then the product $r_{max}.x$ is unitless. The approximation (3) is valid if the product $r_{max}.x$ is very small, relative to 1.

References:

Dolejs, J. 2001. Theory of the age dependence of mortality from congenital defects. Mech. Ageing Dev. 122, 1865-1885.

Dolejs, J. 2003. Analysis of Mortality Decline along with Age and Latent Congenital Defects. Mech. Ageing Dev. 124(5), 679-696.

Dolejs, J. 2016. Single parameter of inverse proportion between mortality and age could determine all mortality indicators in the first year of life. Journal of Theoretical Biology. 397, 193-198.

Dolejs, J. 2017. Modelling Human Mortality from All Diseases in the Five Most Populated Countries of the European Union. The Bulletin of Mathematical Biology. 79(11), 2558–2598 doi:10.1007/s11538-017-0341-y.
