# Supplementary material for: Database of age trajectories of mortality in 110 countries and web application: Data report
Source: Front Public Health. 2022 Jul 29;10:911589. doi: 10.3389/fpubh.2022.911589 (PMC9374568; doi:10.3389/fpubh.2022.911589)
Supplement: Supplementary file 1 [file Data_Sheet_1.zip › ATM_Dolejs/www/TCIR.pdf]

Theory of Congenital Individual Risks (TCIR).

As the present project aimed to describe the dynamics of the relationship between age and mortality, the following definition of the force of mortality at exact age **x** is used:

$$\mu(x) = \lim_{h\to 0}\frac{D(x+h)}{L(x)} = -\frac{dS(x)}{dx}\Big/ S(x) \cong \frac{Di}{Li}\cdot\frac{1}{(Bi-Ai)} \qquad (1),$$

where ***D(x+h)*** is the theoretical number of deaths in a small age interval **[x, x+h)**, the theoretically infinitesimal increment *h* is positive, and age approaches exact age *x* "from the left". ***S(x)*** represents the survival function (percentage of living people at age *x*), which is valid in principle: ***S(x) = 1-F(x)***, where ***F(x)*** is the cumulative distribution function of the probability of death. The empirical value ***Di*** represents the number of deaths in a specific age interval ***[Ai, Bi)***, while ***Li*** represents the size of the population among which the deaths occurred. Changes in ***Li*** within an age interval ***[Ai, Bi)*** are empirically very small when compared with changes in ***Di***. For this reason, the number of living people ***Li*** at age ***Ai*** can be used instead of ***the average number of living people***. In other words, population ***Li*** goes through the "window" in time or through the age interval ***[Ai, Bi)***. The product ***Li•(Bi-Ai)*** in equation (1) represents the number of "***person-years***", or the number of years lived by members of the population between ages ***Ai*** and ***Bi***.

The age trajectory of mortality is assumed to be an unknown theoretical curve, and it is constructed using the right side of equation (1). Mortality rate at an exact age, force of mortality, or simply mortality rate describes different age groups similarly to the way a decay constant describes the force of radioactive decay on different radionuclides. In this comparison, the different radionuclides correspond to the various groups of patients.

Basic assumption in ***TCIR*** is that the decrease of mortality rates with age after the birth is caused by the depletion of individuals with more sever congenital impairment. Possible changes of the impairments with age are negligible if compared with the variability of congenital impairments (congenital anomalies or impairments originated in the perinatal period). Each congenital individual risk ***r*** is assumed to be ***age independent*** if compared with the whole spectrum of congenital impairments in born population. A subpopulation is characterized by the congenital individual risk of death ***r*** and by the initial number of born children $\mathbf{L_o(r)}$. The subpopulation with constant risk of death ***r*** is exponentially reducing during age **x** and number of living at age **x** is:

$$\mathrm{L}(\mathrm{r},\mathrm{x}) = \mathrm{L}_o(r).\exp\left(-r.x\right) \qquad (2)$$

If continuous formalism is used, the number of all living children at age ***x*** is the integral of all **r** values:

$$L(x) = \mathrm{L}_o.S(x) = \int_0^{\propto} \mathrm{L}(\mathrm{r},\mathrm{x}).dr = \int_0^{\propto} \mathrm{L}_o(r).\exp(-r.x)\,.dr \qquad (3)$$

Mortality rate in the whole population in age ***x*** using the assumption (2) is:

$$\mu(\mathrm{x}) = -\frac{\partial \mathrm{S(x)}}{\partial \mathrm{x}}\Big/ \mathrm{S(x)} = -\frac{\partial \mathrm{L(x)}}{\partial \mathrm{x}}\Big/ \mathrm{L(x)} = -\frac{\int_0^{\propto} -\mathrm{r.L_o(r).exp(-r.x).dr}}{\int_0^{\propto} \mathrm{L_o(r).exp(-r.x).dr}} \qquad (4)$$

Since the empirical changes of ***L(x)*** are very small, if compared to the empirical changes of the numbers of deaths ***D(x)*** (the numerator in (4)), the denominator may be replaced by the number of all born children ***Lo***. Empirically, ***L(x)*** varies less than ***2%***, while the numbers of deaths ***D(x)*** ***varies in the magnitude of more than three orders during the first 20 years***. Consequently, the following basic formula is valid:

$$\mu(x) \cong \frac{\int_0^{\propto} r.L_o(r).exp(-r.x).dr}{L_o} = \int_0^{\propto} r.f(r).exp(-r.x)\,.dr \qquad (5),$$

where ***f(r)*** is the density function of ***r*** at the moment of birth. It may be interpreted by the following formula:

$$f(r) = \frac{\mathrm{L}_o(r)}{\mathrm{L}_o} = \frac{\mathrm{L}_o(r)}{\int_0^{\propto}\mathrm{L}_o(r).dr} \qquad (6)$$

The denominator in the equation (6) is the number of all born people $\mathbf{L_o}$.

Mathematically, mortality rate in the whole population at age x is the Laplace transform of the product **r.f(r)** in the equation (5). For example, if ***f(r)*** is the density function of the log-normal distribution or if the rule "**the more severe the impairment, the less frequently it occurs in the born population**" (f (r) ≅ constant/r) is valid, mortality rate is given by:

$$\mu(x) \cong \int_0^{\propto} c.\exp(-r.x)\,.dr = c.\left[\frac{\exp(-r.x)}{-x}\right]_0^{\infty} = \frac{c}{x} \qquad (7)$$

The model is valid for ATTM in all aggregated populations and in the majority of countries. It is also valid for CACNS up to higher ages with higher coefficients of determination.

It was also shown that, if $f(r)$ is the density function of normal distribution with big variation, or if **f(r)** is approximately constant in **an important interval**, mortality rate is given by:

$$\mu(x) \cong \int_0^\infty r.c.\exp(-r.x).dr = c.\left[\frac{-r.\exp(-r.x)}{x} - \frac{\exp(-r.x)}{(-x)^2}\right]_0^\infty = \frac{c}{x^2} \quad (8)$$

This model may describe ATM due to the chapter XVI. "Certain conditions originating in the perinatal period".

What does "**an important interval of r**" mean? The theoretical range of r is from zero to infinite, but it is clear that, empirically, it should range between limited values (r_{min}, r_{max}). For example, if r_{max} is about 1000 and r_{min} is about 0.0001 then the formulas (7) and (8) are numerically valid within the age range **[0, 20)** years (it follows from simple numerical calculations). In other words, the equations (7) and (8) are weakly affected by the majority of the population with very low r, and shape of ATM is determined by small subpopulations with higher values of r. Consequently, the assumptions about distribution of the r value are, in fact, related only to a small part of the born population with higher r. For example, the assumption "**the more severe the impairment, the less frequently it occurs in the born population**" is, in fact, related to the subpopulation with higher r values. This is not true for ATM due to CACNS because the age range for formula (7) is wider and relatively smaller values of r may be important. In a simplified way, **lower ages** correspond to **higher values** of r while **higher ages** correspond to **lower values** of r in the formalisms.

Bending ATM according to *TCIR*.

ATM due to diseases (due to chapters of ICD10) which are not related to congenital impairment (to congenital anomaly or to impairment originating in the perinatal period) are bending. It means slower decrease during the first year and steep decrease after the first year according the formula (7). ATM due to malignant neoplasm (the second chapter of ICD10) are very important exception because they are age independent within the age range from the first month up to the age of 20 years.

The group "**Other diseases**" was created here to demonstrate the phenomenon and it contains fourteen chapters: 1, 3:15. According to TCIR bending ATM are caused by the evidence that in these chapters may contain only small values of r and the ATM are caused by latent congenital impairments.

Formally, if f (r) ≅ constant/r and some maximal limit bounds congenital individual risks in the born population, then the following approximation is valid:

$$\mu(x) = \int_0^{r_{max}} c.e^{(-r.x)}dr = c.\left[\frac{e^{(-r.x)}}{-x}\right]_0^{r_{max}} = c.\frac{e^{(-r_{max}.x)}}{-x} - c.\frac{1}{-x} = \frac{\mu_1}{x}.\left[1 - e^{(-r_{max}.x)}\right] \quad (9)$$

If the product $r_{max}.x$ is small in equation (9), then the theoretical mortality rate is approximately constant according to the following relationship (10):

$$\mu(x) = \frac{\mu_1}{x}.\left[1 - e^{(-r_{max}.x)}\right] \cong \frac{\mu_1}{x}\left[1 - (1 - r_{max}.x)\right] = \mu_1.r_{max} \quad (10)$$

If a time unit of 1 year is used, and if r is represented per year, then the product $r_{max}.x$ is unitless. The approximation (10) is valid if the product $r_{max}.x$ is very small, relative to 1 and it may be valid during the first year after the birth.

If f (r) ≅ constant (or normal distribution with big variation) and some maximal limit bounds congenital individual risks in the born population, then the following approximation is valid:

$$\mu(x) = c.\left[\frac{-r.\exp(-r.x)}{x} - \frac{\exp(-r.x)}{x^2}\right]_0^{r_{max}} = \frac{\mu_1}{x^2}\left[1 - \exp(-r_{max}.x).(1 + r_{max}x)\right] \quad (11)$$

For higher ages the element in brackets equals to one and formula **(8)** is valid (it means for x more than one or two years).
On the other hand, if ages x are smaller and limit of individual risks r_{max} is also smaller (if the product $r_{max}x$ is near zero) then mortality rates are age independent and equals to the constant $\mu_1 r_{max}^2/2$.

References:

Dolejs, J. 2001. Theory of the age dependence of mortality from congenital defects. Mech. Ageing Dev. 122, 1865-1885.

Dolejs, J. 2003. Analysis of Mortality Decline along with Age and Latent Congenital Defects. Mech. Ageing Dev. 124(5), 679-696.

Dolejs, J. 2016. Single parameter of inverse proportion between mortality and age could determine all mortality indicators in the first year of life. Journal of Theoretical Biology. 397, 193-198.

Dolejs, J. 2017. Modelling Human Mortality from All Diseases in the Five Most Populated Countries of the European Union. The Bulletin of Mathematical Biology. 79(11), 2558–2598 doi:10.1007/s11538-017-0341-y.

Dolejs J, Homolkova H, Maresova P. Congenital anomalies of the central nervous system and modeling the decrease of mortality rate with age: WHO metadata from nine European countries. Frontiers in Neurology - Pediatric Neurology. 2018, 9 (585), JUL 19 2018. doi: 10.3389/fneur.2018.00585 https://www.frontiersin.org/articles/10.3389/fneur.2018.00585/full

Dolejs J. Homolkova H. Why Does Child Mortality Decrease With Age? Modeling the Age-Associated Decrease in Mortality Rate Using WHO Metadata From 14 European Countries. Frontiers in Pediatrics. 2020, 8 (703). doi: 10.3389/fped.2020.527811 https://www.frontiersin.org/article/10.3389/fped.2020.527811

Dolejs J. Homolkova H. Why does child mortality decrease with age? Modeling the age-associated decrease in mortality rate using WHO metadata from 25 countries. Frontiers in Pediatrics. 2021, 9: 2296-2360. doi:10.3389/fped.2021.657298 https://www.frontiersin.org/articles/10.3389/fped.2021.657298/abstract
