# Supplementary figures and images for: Database of age trajectories of mortality in 110 countries and web application: Data report
Source: Front Public Health. 2022 Jul 29;10:911589. doi: 10.3389/fpubh.2022.911589 (PMC9374568; doi:10.3389/fpubh.2022.911589)

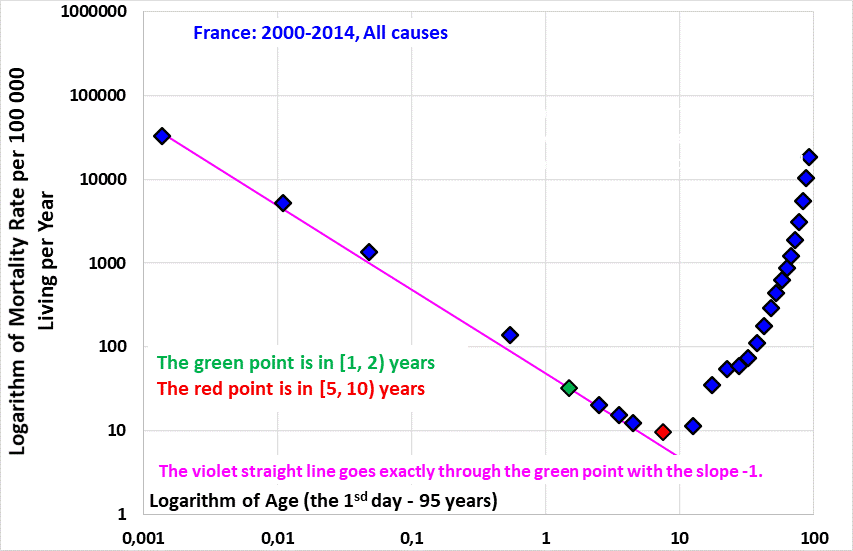

Supplement: Supplementary file 1 [file Data_Sheet_1.zip › ATM_Dolejs/www/ATTM.gif]

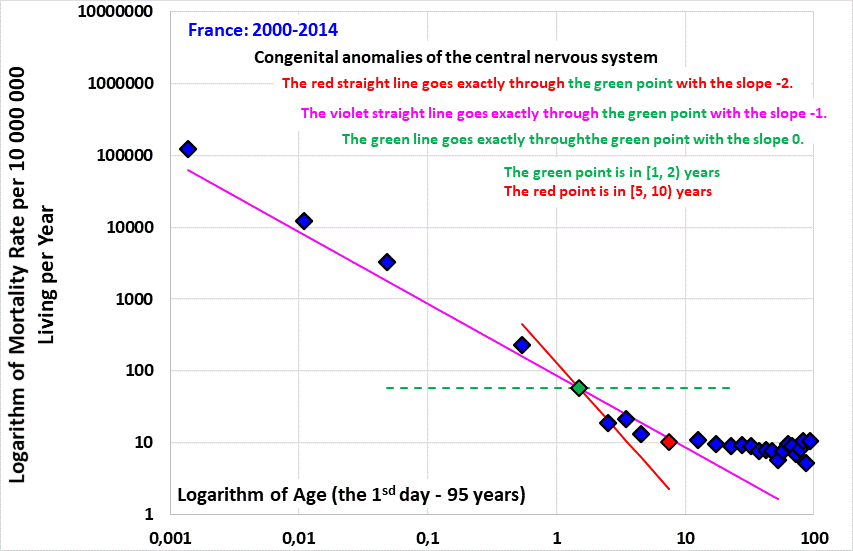

Supplement: Supplementary file 1 [file Data_Sheet_1.zip › ATM_Dolejs/www/CACNS.gif]

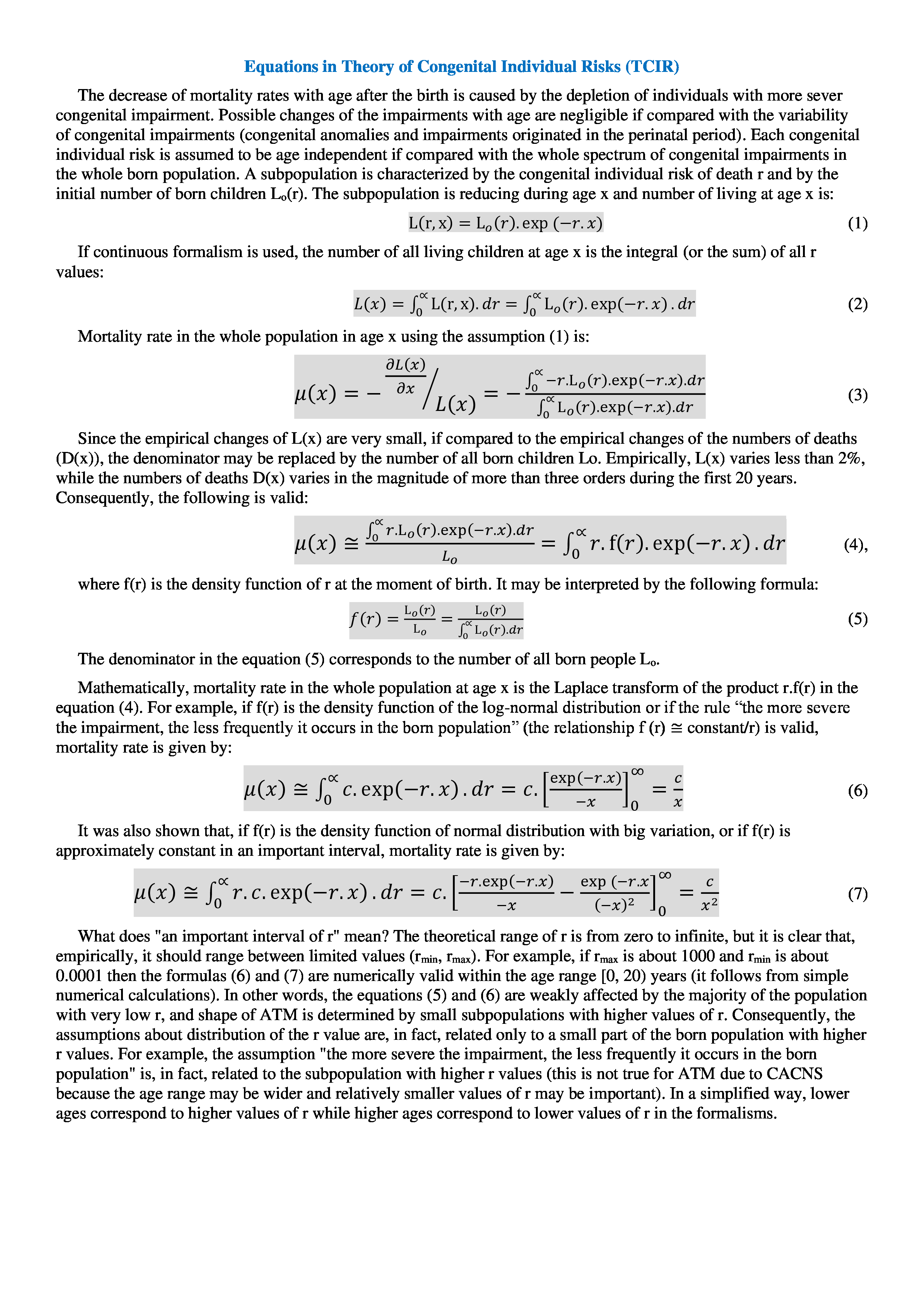

Supplement: Supplementary file 1 [file Data_Sheet_1.zip › ATM_Dolejs/www/Equations1.gif]

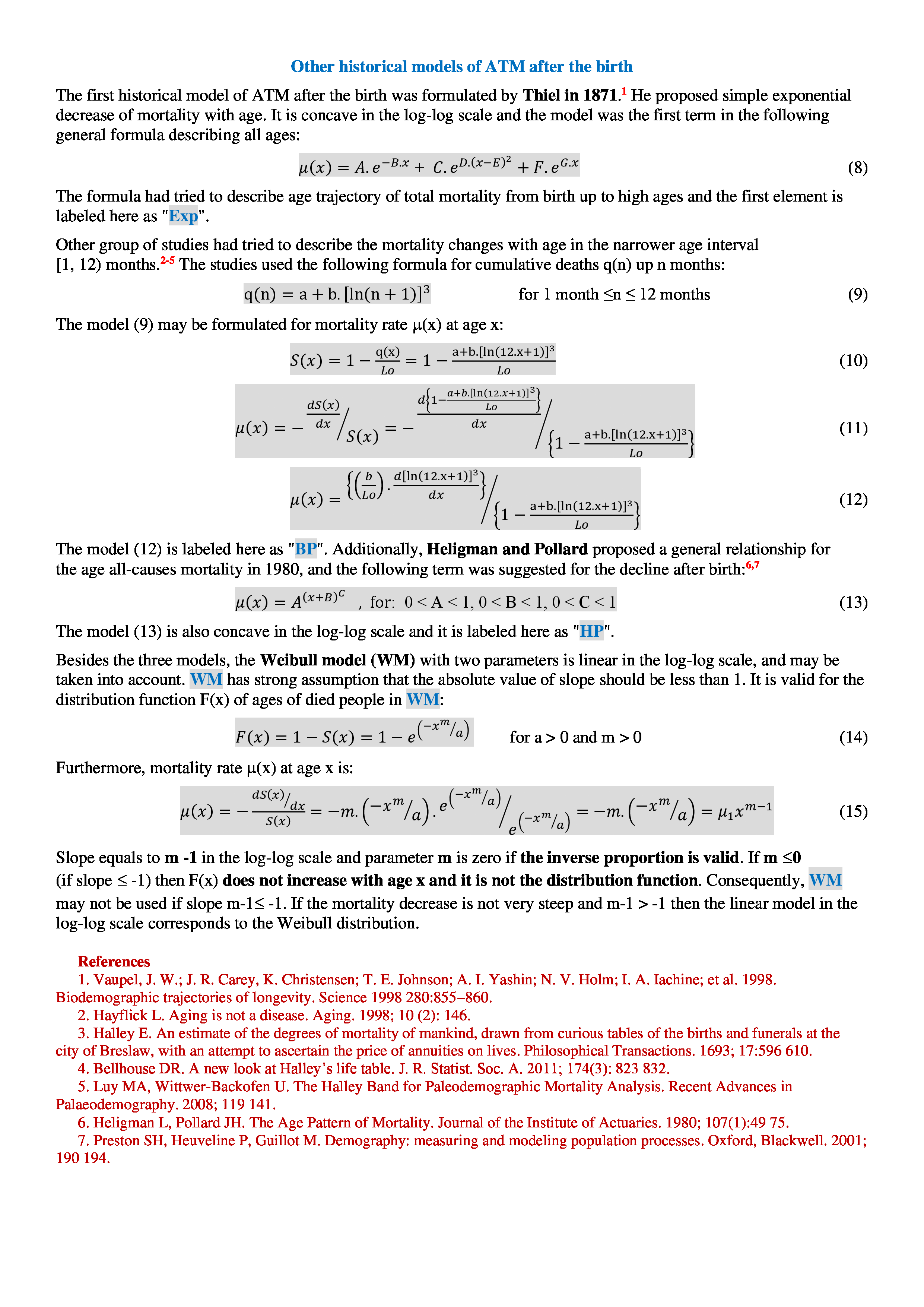

Supplement: Supplementary file 1 [file Data_Sheet_1.zip › ATM_Dolejs/www/Equations2.gif]

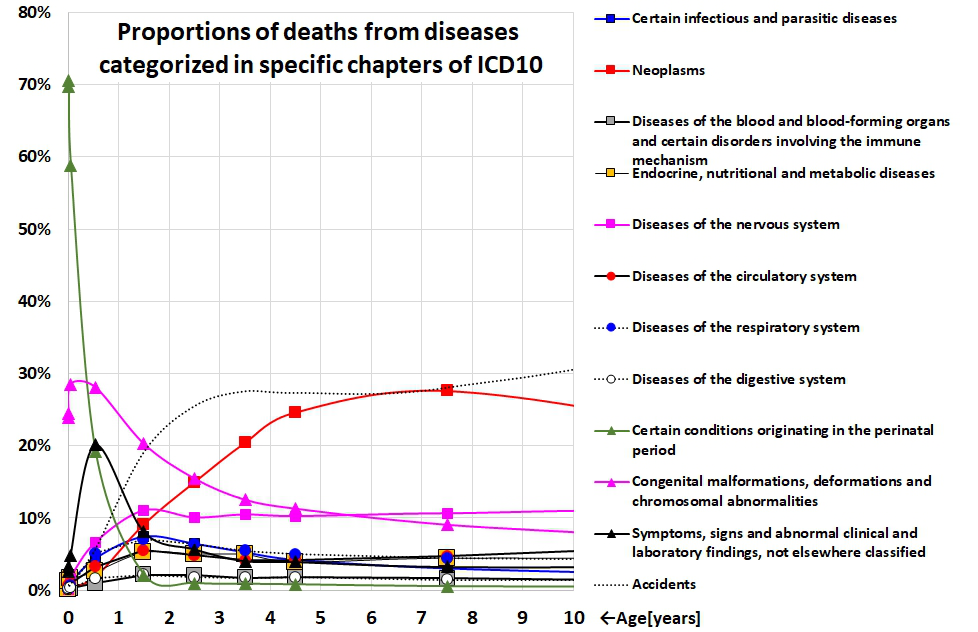

Supplement: Supplementary file 1 [file Data_Sheet_1.zip › ATM_Dolejs/www/F1.png]

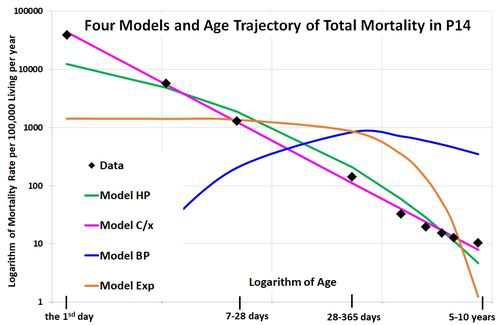

Supplement: Supplementary file 1 [file Data_Sheet_1.zip › ATM_Dolejs/www/F2.png]

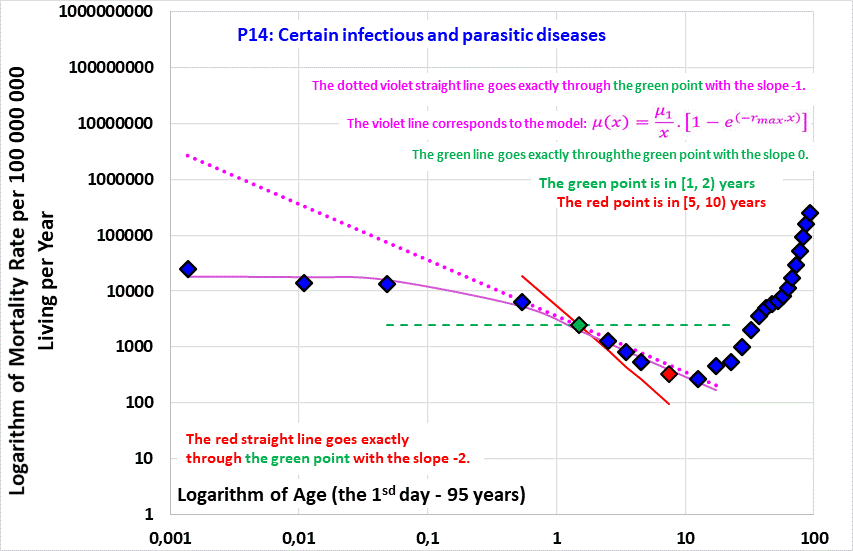

Supplement: Supplementary file 1 [file Data_Sheet_1.zip › ATM_Dolejs/www/FF3.gif]

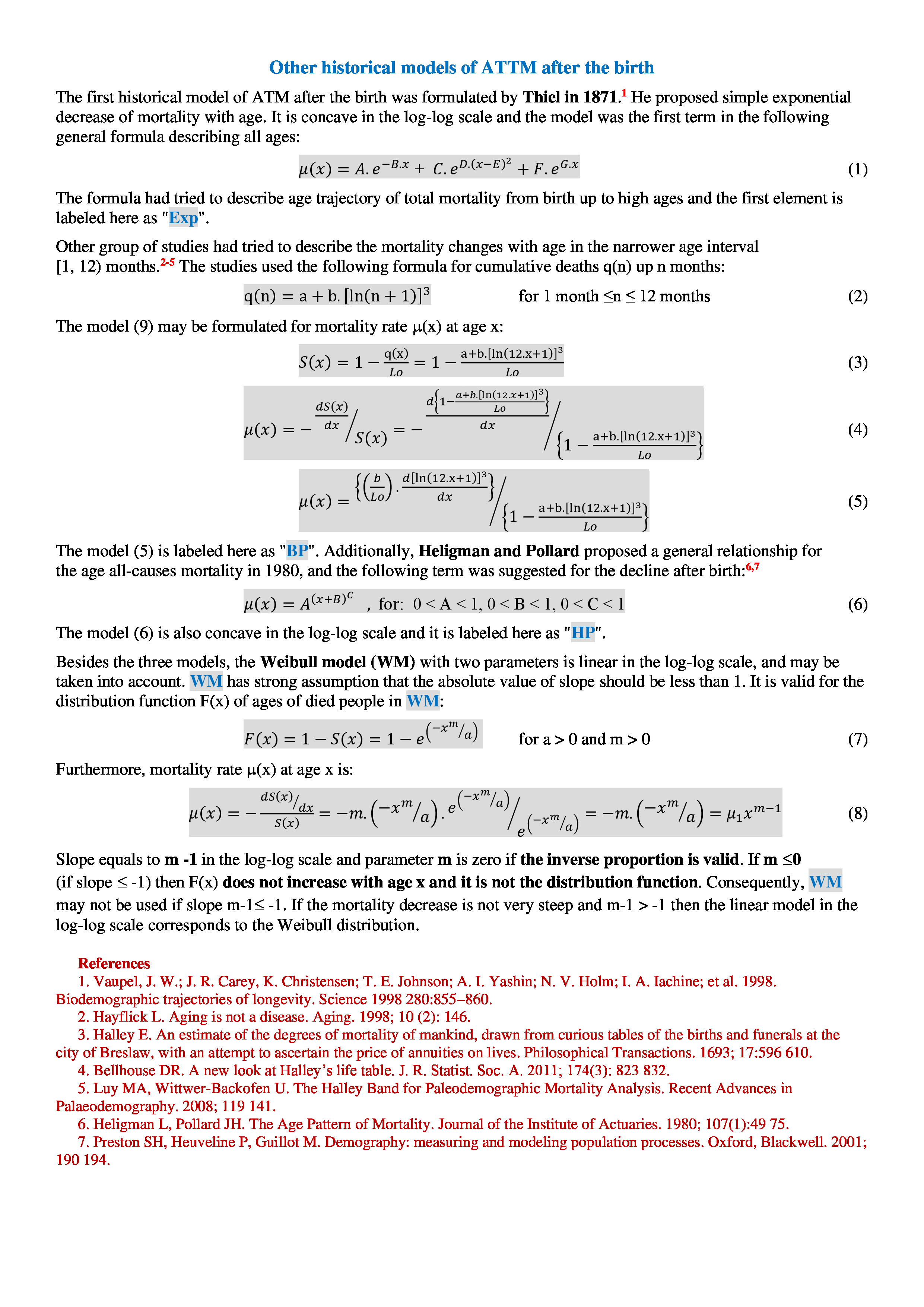

Supplement: Supplementary file 1 [file Data_Sheet_1.zip › ATM_Dolejs/www/Historical models.gif]

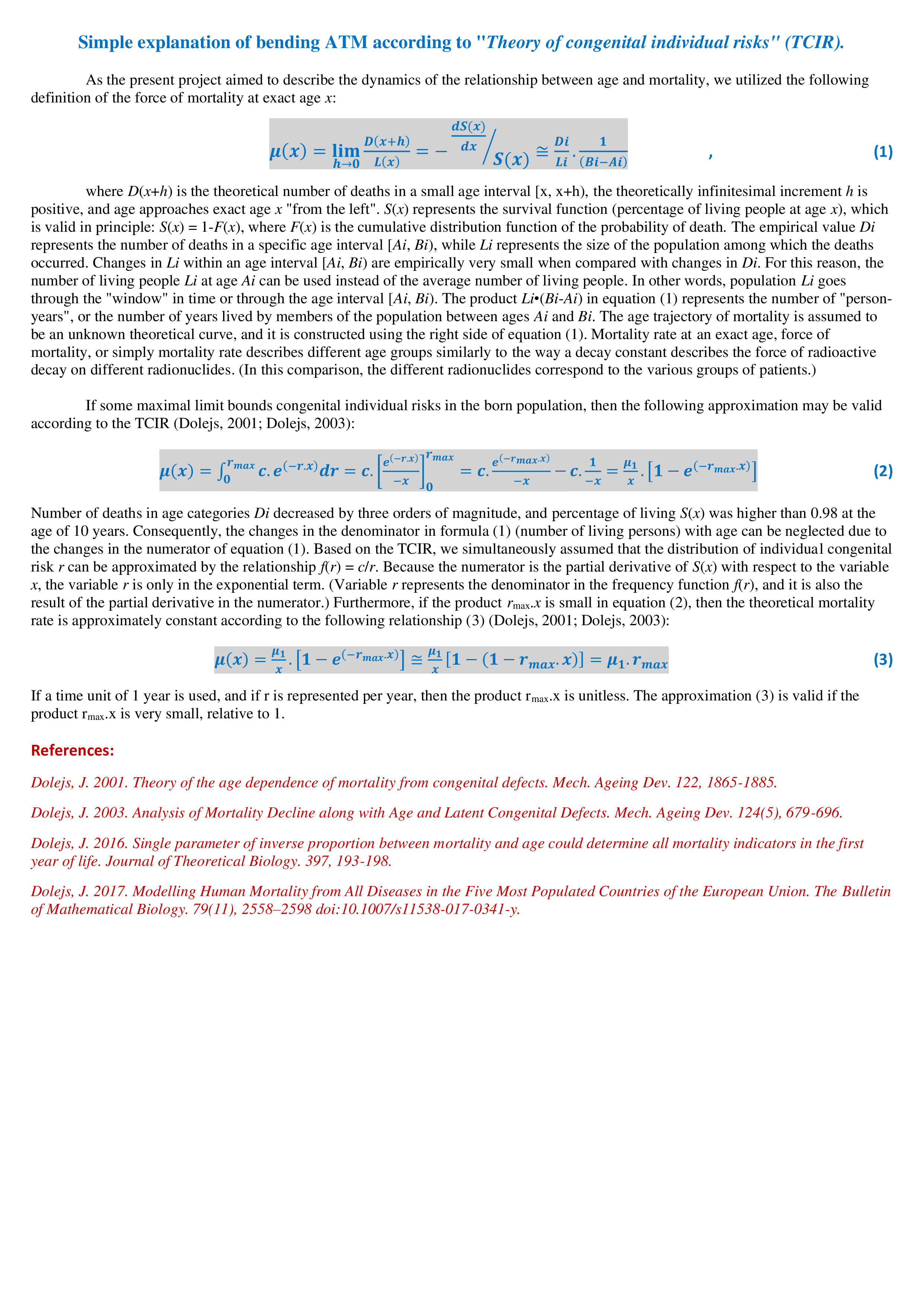

Supplement: Supplementary file 1 [file Data_Sheet_1.zip › ATM_Dolejs/www/Relationships describing bending ATM.gif]

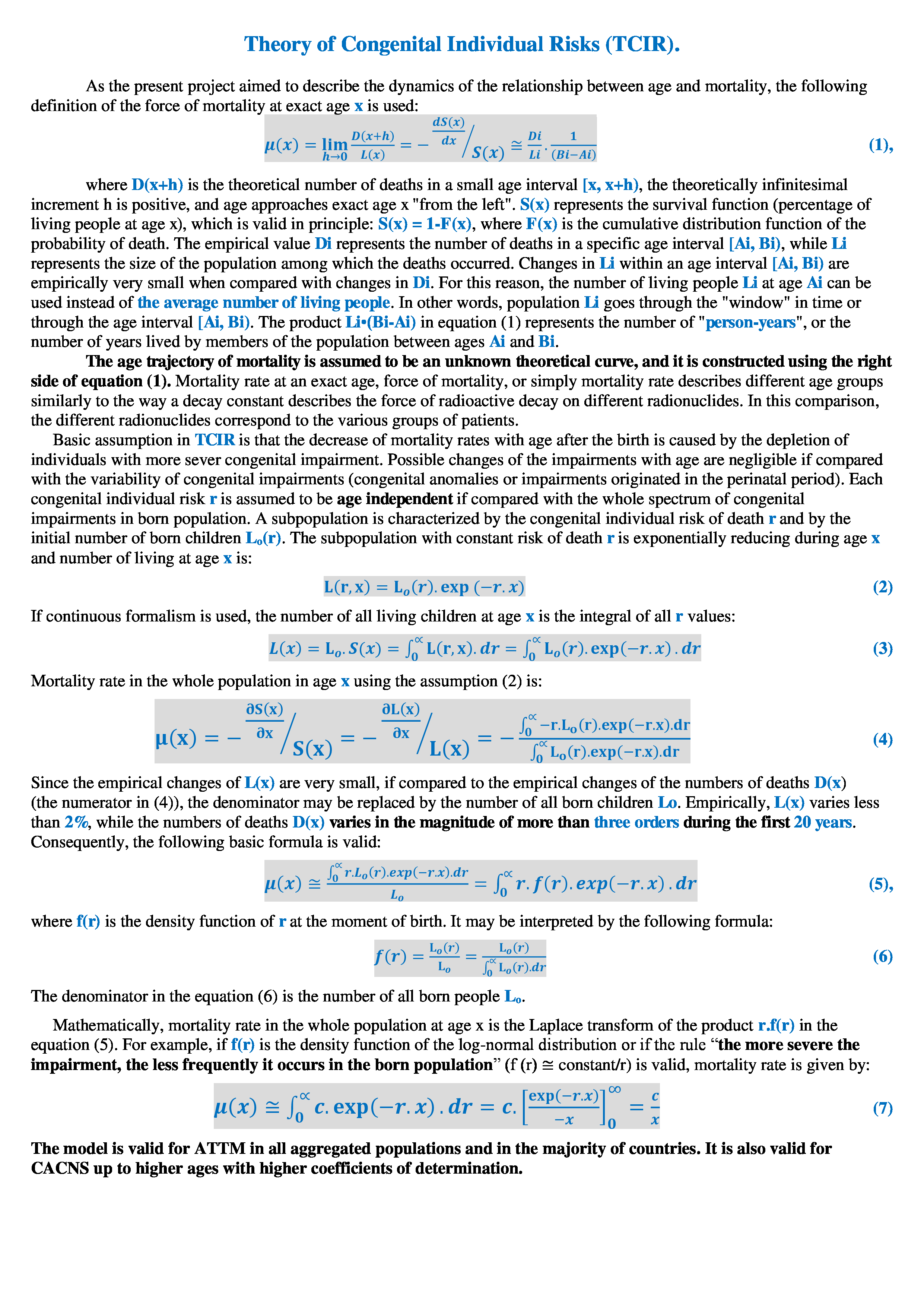

Supplement: Supplementary file 1 [file Data_Sheet_1.zip › ATM_Dolejs/www/TCIR1.gif]

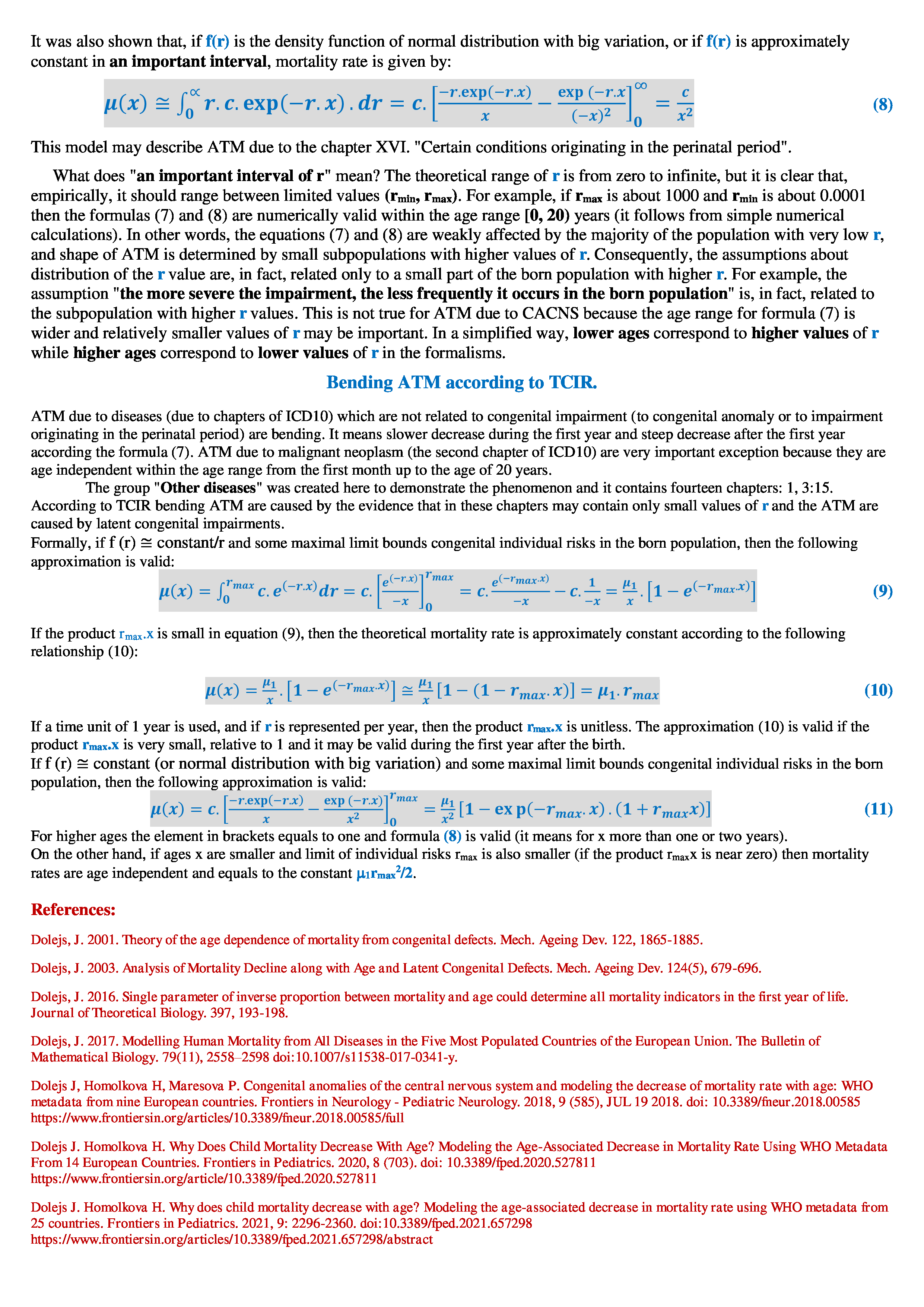

Supplement: Supplementary file 1 [file Data_Sheet_1.zip › ATM_Dolejs/www/TCIR2.gif]
